# Elevated Circulating Fetuin-B Levels Are Associated with Insulin Resistance and Reduced by GLP-1RA in Newly Diagnosed PCOS Women

**DOI:** 10.1155/2020/2483435

**Published:** 2020-09-30

**Authors:** Mani Mokou, Shan Yang, Bin Zhan, Shan Geng, Kejia Li, Mengliu Yang, Gangyi Yang, Wuquan Deng, Hua Liu, Dongfang Liu, Zhiming Zhu, Ling Li

**Affiliations:** ^1^Key Laboratory of Diagnostic Medicine (Ministry of Education) and Department of Clinical Biochemistry, College of Laboratory Medicine, Chongqing Medical University, 400016 Chongqing, China; ^2^Department of Endocrinology, The Second Affiliated Hospital, Chongqing Medical University, Chongqing 400010, China; ^3^The Thirteenth People's Hospital of Chongqing, Chongqing 400016, China; ^4^Department of Endocrinology, Chongqing University Central Hospital, Chongqing Emergency Medical Center, Chongqing 400014, China; ^5^Department of Pediatrics, University of Mississippi Medical Center, 2500 North State Street, Jackson, MS 39216-4505, USA; ^6^Department of Hypertension and Endocrinology, Daping Hospital, Army Medical University, Chongqing Institute of Hypertension, Chongqing 400010, China

## Abstract

**Background:**

Previous studies have suggested that Fetuin-B seems to be a secreted adipokine related to metabolic diseases. However, the results have been inconsistent. Here, our objective is to investigate the changes in circulating Fetuin-B levels in women with polycystic ovary syndrome (PCOS) and analyze the association of Fetuin-B and insulin resistance (IR).

**Methods:**

The current study is comprised of a cross-sectional study and a series of interventional studies. Oral glucose tolerance test (OGTT) and euglycemic-hyperinsulinemic clamp (EHC) were engaged to assess glucose tolerance and insulin sensitivity. Serum Fetuin-B levels were determined by ELISA.

**Results:**

Serum Fetuin-B and TNF-*α* levels were markedly increased in women with PCOS compared to healthy women. Circulating Fetuin-B was positively associated with body mass index, waist-to-hip ratio, the percentage of body fat (FAT%), systolic blood pressure, triglyceride, low-density lipoprotein cholesterol, fasting blood glucose, 2 h blood glucose after glucose overload, fasting insulin, 2 h insulin after glucose overload, HOMA-insulin resistance index (HOMA-IR), the area under the curve for insulin (AUCi), AUCg, and TNF-*α*, while negatively associated with *M* value and follicular stimulating hormone (FSH). During the EHC, Fetuin-B levels were found to be significantly increased in PCOS women. After a glucose challenge, serum Fetuin-B levels in healthy women were significantly increased. Lipid infusion reduced serum Fetuin-B levels in 30 healthy subjects. After six months of glucagon-like peptide-1 receptor agonist (GLP-1RA) intervention, serum Fetuin-B concentrations in PCOS women markedly decreased following ameliorated IR.

**Conclusion:**

Our results indicate that Fetuin-B may be a biomarker of IR in individuals with PCOS. This trial is registered with ChiCTR-IIR-16007901.

## 1. Introduction

Polycystic ovary syndrome (PCOS) is a relatively common endocrine disease in women of reproductive age. It is characterized by ovulation dysfunction, polycystic ovarian changes, hyperandrogenism, and/or clinical manifestations of hyperandrogenism [1]. PCOS is related to a variety of diseases, including low-grade inflammatory state, insulin resistance (IR), metabolic syndrome (MetS), obesity, dyslipidemia, and hypertension. The epidemiological investigation has shown that nearly 70% of women with PCOS have IR [2, 3]. These conditions can eventually develop into type 2 diabetes mellitus (T2DM) [4–6]. Although the pathogenesis of PCOS is not clear, IR, hyperinsulinemia, and hyperandrogenism are capital elements related to the occurrence and development of PCOS.

The liver has a key role in the metabolism of glucose and lipid *in vivo*. It is also a source of many hormones, which are often called hepatokines [7]. Fetuin-A and Fetuin-B belong to the Fetuin family and are liver-derived cytokines [8]. It has been reported that there are 22% sequence similarities between Fetuin-A and Fetuin-B in mice and humans. In humans, mice, and rats, Fetuin-B protein has a 61% amino acid homology [9]. Recently, in an animal study, Fetuin-B was found to impair glucose tolerance (IGT) and insulin signaling, while Fetuin-B knockdown ameliorated glucose tolerance [10]. In human studies, circulating Fetuin-B levels were elevated in obese individuals, patients with gestational diabetes mellitus (GDM) and T2DM [11, 12], and related to IR and insulin secretion [10, 12]. In patients with nonalcoholic fatty liver disease (NAFLD), serum Fetuin-B levels increased or remained unchanged [8, 11, 13–15]. Taken together, previous studies suggested that Fetuin-B seemed to be an adipokine or hepatokine related to metabolism.

In this study, we first investigated serum Fetuin-B concentrations in healthy individuals and newly diagnosed PCOS women with and without IR. Second, we explored the association of serum Fetuin-B with IR using multiple intervention experiments, including a euglycemic-hyperinsulinemic clamp (EHC, euglycemic-hyperinsulinemic state), an oral glucose tolerance test (OGTT) (elevated blood glucose and insulin levels), or liraglutide, a glucagon-like peptide-1 receptor agonists (GLP-1RA) treatment (from insulin-resistant to insulin-sensitizing state) in healthy controls and/or women with PCOS.

## 2. Materials and Methods

### 2.1. Cross-Sectional Study

Three hundred ninety-eight women include 257 women with newly diagnosed PCOS (nPCOS) and 141 healthy women participated in the study (Figure S1). PCOS patients were recruited from outpatients attending the Department of Endocrinology at the Second Affiliated Hospital, Chongqing Medical University, Chongqing, China. PCOS patients were diagnosed by the revised 2003 Rotterdam European Society of Human Reproduction and Embryology (ESHRE)/American Society of Reproductive Medicine (ASRM) PCOS Consensus Workshop Group diagnostic criteria [1]. Diagnostic and exclusion criteria were detailed in the Supplementary data. One hundred forty-one healthy women with regular menstruation were recruited as the control individuals from the community, schools, and universities through advertisement, or routine medical check-up. Written informed consent was given to all participants. This study was conducted by the Declaration of Helsinki and supported by the ethical committee of Chongqing Medical University.

### 2.2. EHC and OGTT

EHCs were performed in 243 patients with PCOS and 102 healthy women, as previously reported [16]. Briefly, after overnight fasting, a venous catheter was inserted into the antecubital vein for insulin and glucose infusion. Another catheter was implanted into the dorsal vein of the contralateral hand for a blood sample. Human insulin (1 mU/kg/min) was infused for two hours, and 20% glucose was infused to maintain blood glucose at the basal levels. During the EHC, blood glucose was measured every 15 min for guiding the rate of glucose infusion (GIR). Glucose disposal rate (GRd) equaled to GIR during the clamp steady-state and was related to body weight (*M* value). To determine circulating Fetuin-B levels and other parameters, blood samples were taken at indicated times (0, 80, 100, and 120 min). The OGTT was detailed in the supplementary data. Blood samples were centrifuged to separate serum and stored at -80°C for subsequent analysis.

### 2.3. Lipid Infusion Study for FFA-Induced IR

**Figure 1:**
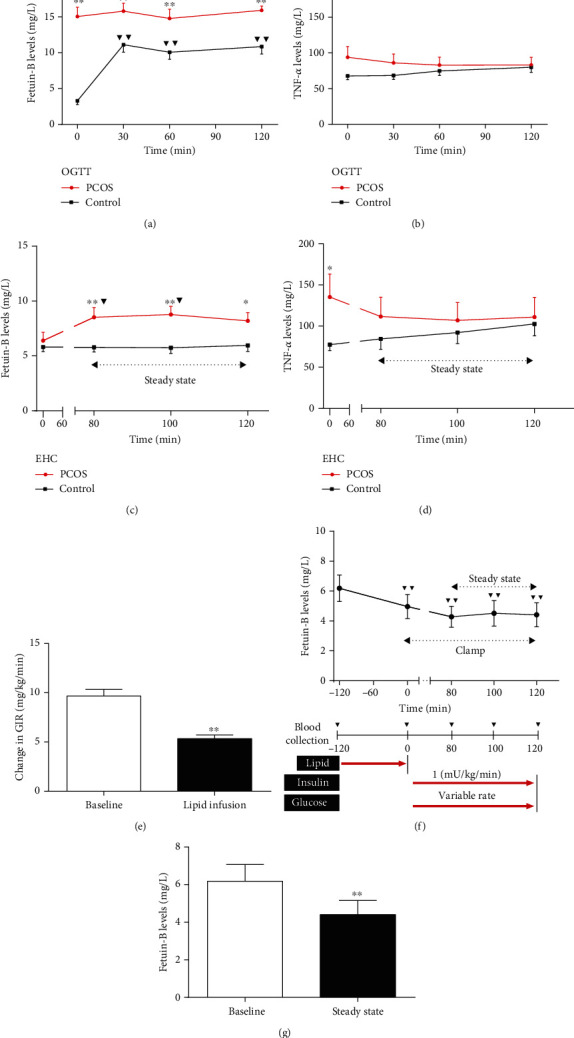
Circulating Fetuin-B concentrations in interventional studies. (a) Circulating Fetuin-B concentrations in healthy and PCOS women during an OGTT. (b) Circulating TNF*α* levels in PCOS and healthy women during an OGTT. (c) Time course of circulating Fetuin-B changes in healthy and PCOS women during the EHC. (d) Time course of circulating TNF*α* changes in healthy and PCOS women during the EHC. (e) Glucose infusion rates (GIR) in control women at pre- and postlipid infusion. (f) Time course of circulating Fetuin-B changes in control women during lipid infusion combined with EHC. (g) Circulating Fetuin-B levels at pre- and postlipid infusion. Data are means ± SEM. ^∗^*p* < 0.05 or ^∗∗^*p* <  0.01 vs. control or baseline; ▼*p* < 0.05 or ▼▼*p* < 0.01 vs. baseline.

To investigate the impact of free fatty acid- (FFA-) induced acute IR on circulating Fetuin-B *in vivo*, a lipid infusion was performed in 30 healthy and young adults [17]. Following a 12 h overnight fast, participants were given a 20% intralipid/heparin (0.4 units/kg/min; Pharmacia and Upjohn, Milan, Italy) infusion at a constant rate (1.5 mL/min) for 4 hours. 120 min after the start of the lipid infusion, a 120 min EHC was performed as described previously [17]. The experimental protocol was shown in Figure 1. Venous blood was collected before and at the indicated time during the experiment.

### 2.4. Biochemical Parameters and Hormone Measurements

Biochemical parameters and hormone measurements were detailed in the supplementary data [17].

### 2.5. Measurements of Serum Fetuin-B and Tumor Necrosis Factor-Alpha (TNF*α*)

Serum Fetuin-B was measured with a commercial ELISA Kit (Catalog: ELH-FetuinB; RayBiotech, Inc. Norcross, GA, USA). The sensitivity of the Kit was 4.0 ng/mL. As the manufacturer's instructions, the intra- and interassay coefficients of variation (CV) were 10% and 12%, respectively. Also, serum TNF*α* levels were measured by an ELISA kit (4A biotech Co, Beijing, China). The detection limit for this measurement was 7 pg/mL. Both the intra- and inter-CV were 10%, as previously reported [18].

### 2.6. Intervention Study of GLP-1RA

A study schematic diagram was presented in Figure S2. A total of 36 women with PCOS attended this self-controlled clinical study of GLP-1RA treatment without a placebo-controlled group. PCOS women were given liraglutide, a GLP-1RA. Liraglutide dose was increased weekly by 0.6 mg/d until using 1.8 mg once daily before breakfast for 24 weeks. Inclusion criteria were age 18-35 year and BMI > 25 kg/m^2^. Women who were pregnant or intended to be pregnant were not included in the study. These women were requested to adhere to the prestudy lifestyle and dietary habits throughout the study. All individuals were given informed written consent about the side effects of GLP-RA at the onset of therapy. Participants were interviewed at the beginning of the study, at weeks 12 and 24. At each interview, OGTT and EHC were performed to assess IR, and serum Fetuin-B, hormone, and biochemical parameters were measured (Figure S2). These women underwent anthropometric examinations at pretherapy, week 12, and week 24. Blood samples were obtained for Fetuin-B and various other parameters at 0800 hours on day 0 pretherapy and on day one at weeks 12 and 24. A flowchart for cross-sectional and interventional studies was shown in Figure S1.

### 2.7. Anthropometric Examination

These contents were detailed in the Supplementary data.

### 2.8. Calculations

The free androgen index (FAI) = [testosterone (TEST)/sex hormone - binding globulin (SHBG) × 100], as previously reported [17]. Computer software (HOMA Calculator v2.2.2) was used to calculate the homeostasis model assessment of IR (HOMA2-IR) [19]. *M* value was calculated by GIR/body weight, as previously described [17]. The cut-off point for IR was defined as *M* value < 6.28 [20].

### 2.9. Statistical Analysis

Statistical Package for SPSS 19.0 was used to perform all analyses. Data were expressed as means ± SD or mean ± SE. Variables with a nonnormal distribution were transformed by logarithm or square roots before analysis. ANOVA, paired or unpaired *t*-test were used to perform the comparison between groups. The differences between the two groups were analyzed with the Mann-Whitney *U* test for nonnormally distributed independent variables. The relationship between serum Fetuin-B and other parameters was analyzed by simple and multiple regression analyses. Binary logistic regression analysis was used to examine the associations of Fetuin-B with PCOS. The distribution of Fetuin-B was divided into tertiles, and the row mean scores and the Cochran-Armitage trend tests were used to estimate the significant trends across increasing tertiles. All of the statistical analyses were two-sided, and *p* < 0.05 was considered significant.

## 3. Results

### 3.1. Hormone, Anthropometric, and Biochemical Parameters in PCOS and Healthy Women

Table 1 showed baseline characteristics and hormonal concentrations in healthy and PCOS women. Age, high-density lipoprotein cholesterol (HDL-C), FFAs, luteinizing hormone (LH), estradiol, prolactin, and progesterone (Prog) were similar in the study population. In PCOS women, body mass index (BMI); waist-to-hip ratio (WHR); the percentage of body fat (Fat %); blood pressure; blood fat, including total cholesterol (TC), triglyceride (TG), and low-density lipoprotein cholesterol (LDL-C); glucose metabolic-related parameters including fasting blood glucose (FBG), 2 h postglucose load blood glucose (2 h-BG), and the area under the curve for glucose and insulin (AUC); and IR-related parameters including fasting insulin (FIns), 2 h insulin after glucose overload (2 h-Ins), and HOMA-IR were significantly higher, whereas *M* value was lower when compared with healthy women. As shown in Table 1, PCOS women had substantially higher dehydroepiandrosterone-sulfate (DHEA-S) and TEST, whereas follicle-stimulating hormone (FSH) and SHBG were lower compared with the control women. In addition, PCOS women had higher FAI relative to healthy women (Table 1). These results suggest that there might be IR and abnormal sex hormone secretion in PCOS patients.

### 3.2. Elevated Fetuin-B Concentrations in Women with PCOS and the Association of Serum Fetuin-B with Other Variables in the Study Population

**Figure 2:**
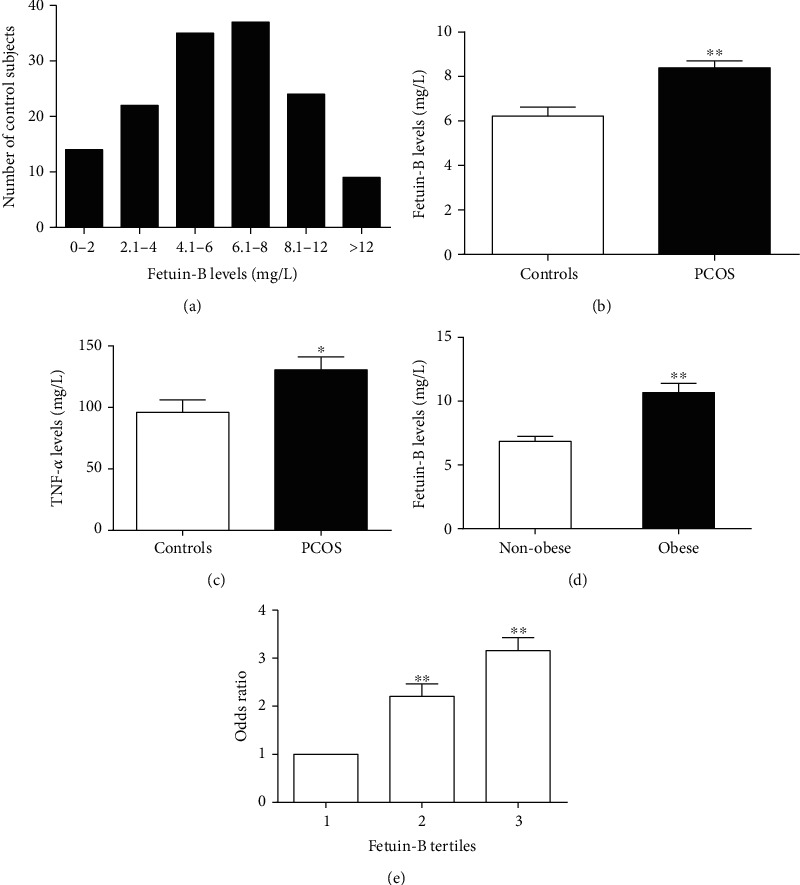
Concentrations of serum Fetuin-B in the study population. (a) Distribution of serum concentration of Fetuin-B in 141 control women. (b) Circulating Fetuin-B levels in PCOS and control women. (c) Circulating TNF*α* levels in PCOS and control women. (d) circulating Fetuin-B levels according to BMI (lean: BMI < 24 kg/m^2^; obese: BMI ≥ 28  kg/m^2^). (e) Prevalence of elevated PCOS in different quartiles of Fetuin-B. Data are means ±  SEM. ^∗^*p* < 0.05 or ^∗∗^*p* < 0.01 vs. controls, nonobese, or tertile 1.

The distribution of Fetuin-B concentrations in 141 healthy women was shown in Figure 2(a). The range of circulating Fetuin-B in these individuals (age 18-35 years) was between 5.41 and 9.01 mg/L (90%). As shown in Figure 2(b), serum Fetuin-B levels were significantly increased in PCOS individuals than those in controls. This increase remained marked after adjustment for age and BMI (Table 1). In addition, a higher serum TNF*α* concentration was found in women with PCOS compared with those control individuals, suggesting a low-grade inflammatory state (Figure 2(c)). Further subgroup analysis showed that circulating Fetuin-B levels were significantly higher in obese PCOS women than those in lean PCOS women (Figure 2(d)). We next performed simple and multiple regression analyses to assess the relationship of serum Fetuin-B with other parameters. We found that in all study population, circulating Fetuin-B was positively associated with BMI, WHR, FAT%, systolic blood pressure (SBP), TG, LDL-C, FBG, 2 h-BG, FIns, 2 h-Ins, HOMA-IR, AUCi, AUCg, and TNF*α*, while negatively associated with *M* value (Table 2). Furthermore, circulating Fetuin-B had a significant negative correlation with FSH (Table 2). Even after adjusting for WHR and age, these correlations were still statistically significant. We also performed multiple regression analyses in the study population and found that only WHR and *M* value were independent related to serum Fetuin-B (Table 2). The multiple regression equation was as follows: *Y*_Fetuin−B_ = 1.324–0.400 *X*_*M* value_ + 0.204 *X*_WHR_ (*R*^2^ = 0.302, *p* < 0.01).

Furthermore, multivariate logistic regression analysis showed that serum Fetuin-B was significantly associated with the incidence of PCOS. This relationship remains to exist, even after controlling for other parameters and lipid profile (Table S1). When serum Fetuin-B concentrations were analyzed by row mean scores and Cochran-Armitage trend test, decreasing concentrations led to a significant linear trend and were independently related to IR and PCOS (Table S2). We further divided serum Fetuin-B concentrations into three tertiles in all study populations (tertile 1, <5.47 mg/L, *n* = 134; tertile 2, 5.48-8.77 mg/L, *n* = 136; tertile 3, >8.77 mg/L, *n* = 128). The tertile 3 of serum Fetuin-B concentration had the highest risk of developing PCOS. The odds ratio was 3.16, as shown in Figure 2(e).

### 3.3. The Predictive Values of Serum Fetuin-B for Predicting PCOS and IR

To explore whether Fetuin-B can predict the occurrence of PCOS and IR, we performed the analysis of receiver operator characteristic (ROC) curves in all study individuals. Based on this analysis, we found that the area under the ROC curves for PCOS (AUC_PCOS_) was 0.73 (95% confidence Interval: 0.675-0.789) with a sensitivity of 76% and a specificity of 61% (Figure S3A, *p* < 0.01). AUC for IR (AUC_IR_) was 0.77 (95% confidence interval: 0.715-0.826) with a sensitivity of 78% and a specificity of 65% (Figure S3B, *p* < 0.01). The best cutoff values for serum Fetuin-B to detecting PCOS and IR were 6.44 and 6.47 mg/L, respectively.

### 3.4. Serum Fetuin-B Concentration in Response to Glucose Challenge in PCOS and Control Women

To investigate the impact of hyperglycemia or/and hyperinsulinemia on serum Fetuin-B levels *in vivo*, we performed an OGTT test in both PCOS and healthy women. After a glucose challenge, serum Fetuin-B levels in healthy women were significantly increased to its highest point within 30 min and then maintained for 90 min (Figure 1(a)), whereas in PCOS women, serum Fetuin-B concentrations remain unchanged during the OGTT (Figure 1(a)). In addition, serum TNF*α* levels remain unchanged in response to glucose challenge in both PCOS and healthy women (Figure 1(b)). These results suggest that hyperglycemia or/and hyperinsulinemia may stimulate Fetuin-B secretion and release into circulation in control women, but not in PCOS women.

### 3.5. Effects of Hyperinsulinemia on Serum Fetuin-B in PCOS and Control Women

To further study whether serum Fetuin-B concentration is affected by the state of euglycemic-hyperinsulinemia, serum Fetuin-B levels were measured in thirty healthy women and thirty women with PCOS during the EHC. During the EHC, insulin infusion resulted in increased insulin concentrations (from 8.0 ± 3.0 to 105.6 ± 23.1 mU/L in healthy subjects and from 19.5 ± 4.9 to 90.1 ± 21.3 mU/L in PCOS patients). An infusion of 25% glucose was given to maintain blood glucose at normal levels (4.5–5.5 mmol/L). At the stable-state of the clamp, PCOS women had a lower *M* values compared with those of healthy women (4.42 ± 1.71 vs. 9.97 ± 2.86 mg/kg per min; *p* < 0.01), indicating the presence of IR in PCOS women. In healthy women, circulating Fetuin-B concentrations were unchanged during the EHC. Importantly, in response to increased insulin levels, serum Fetuin-B concentrations were markedly increased in PCOS females from 6.40 ± 0.75 to 8.52 ± 0.89 mg/L during the clamp (*p* < 0.05 vs. 0 min, Figure 1(c)). This concentration was maintained from 80 min to 100 min of the EHC (Figure 1(c)). Furthermore, serum TNF*α* levels had no difference between healthy and PCOS women during the stable period of the clamp (Figure 1(d)).

### 3.6. Effect of FFA-Induced IR on Serum Fetuin-B In Vivo

To investigate the impact of FFA-induced IR on serum Fetuin-B levels *in vivo*, we next performed lipid infusion combined with an EHC in 30 control individuals (Figure 1(f)). During the steady-state of the clamp, blood glucose levels were maintained at 4.5-5.5 mmol/L. After lipid infusion, the GIR was significantly lower than that before lipid infusion (5.36 ± 0.36 vs. 9.67 ± 0.69 mg/kg/min, *p* < 0.01, Figure 1(e)), indicating an IR induced by FFA in these individuals. During the EHC, lipid infusion resulted in a significant decrease in serum Fetuin-B compared with basal levels (from 6.19 ± 0.89 to 4.96 ± 0.81, then to 4.51 ± 0.86, and finally, to 4.40 ± 0.76 mg/L, *p* < 0.01). Ultimately, this concentration was maintained until the end of the clamp (Figure 1(f)). During lipid perfusion, the average concentration of Fetuin-B was shown in Figure 1(g).

### 3.7. Effects of GLP-1RA Intervention on Serum Fetuin-B Concentrations in PCOS Women

**Figure 3:**
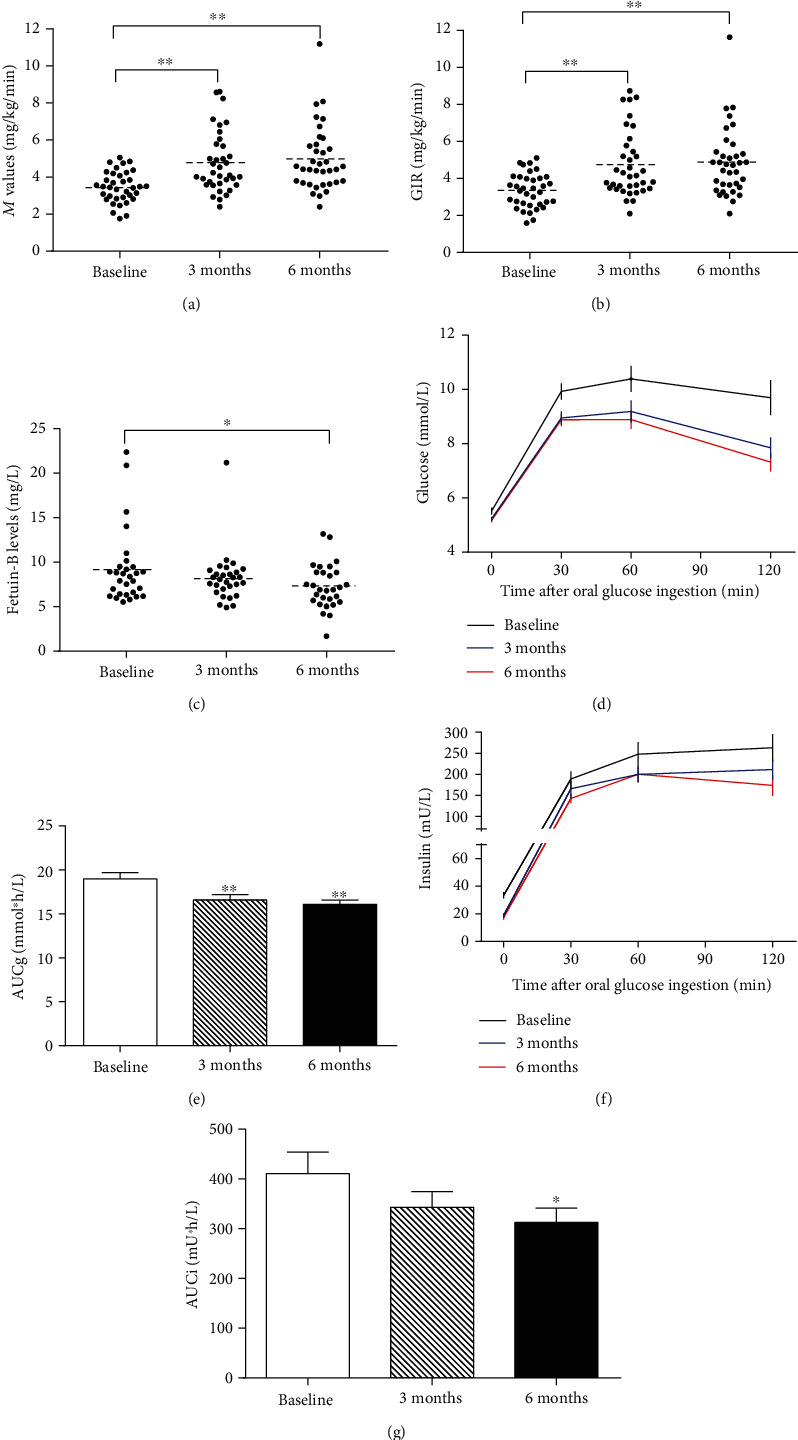
Effects of GLP-1RA treatment on circulating Fetuin-B and insulin sensitivity in PCOS women. (a) *M* values in PCOS subjects during the EHC after GLP-1RA treatment. (b) Average GIR in PCOS subjects during the EHC at pre- and posttreatment. (c) Serum Fetuin-B levels in PCOS subjects before and after GLP-1RA treatment. (d) Blood glucose 572 levels in PCOS subjects during an OGTT at pre- and posttreatment. (e) AUCg in PCOS subjects during an OGTT at pre- and posttreatment. (f) Insulin levels in PCOS subjects during an OGTT at pre- and posttreatment. (g) AUCi in PCOS subjects during an OGTT at pre- and posttreatment. Data are means ± SEM.^∗^*p* < 0.05 or ^∗∗^*p* < 0.01 vs. baseline.

Thirty-six women with PCOS received liraglutide intervention for six months. The biochemical, hormonal, and other parameters pre- and posttreatment were shown in Table S3. Three and six months after GLP-1RA intervention, BMI, FAT %, TG, TC, 2-hBG, FIns, HOMA-_IR_, AUCg, TEST, and FAI were significantly reduced compared with pretreatment (Table S3), whereas GIR and *M* value were significantly increased (Figures 3(a) and 3(b)). Besides, during the OGTT, blood glucose and insulin levels decreased in 60 and 120 minutes after GLP-1RA intervention for 6 months (Figures 3(d) and 3(f)). Furthermore, AUCg and AUCi were also lower after treatment (Figures 3(e) and 3(g)). These results suggest that GLP-1RA intervention resulted in an amelioration in glucose metabolism and IR. Importantly, after three months treatment with GLP-1RA, serum Fetuin-B concentrations in PCOS women showed a downward trend, while after six months of GLP-1RA treatment, serum Fetuin-B levels were markedly decreased (from 9.22 ± 0.77 at pretreatment to 8.23 ± 0.54 at posttreatment three months, and finally to 7.27 ± 0.46 mg/L, *p* < 0.05 for posttreatment six months vs. pretreatment; Figure 3(c)).

## 4. Discussion

Fetuin-B was mainly expressed in the liver and circulates at relatively high levels (*μ*g/mL). However, its exact function and association with glucose metabolism and IR were not completely clear because the previous results were inconsistent. All previous studies were mostly descriptive, superficial, noninterventional, and lacking in sufficient and in-depth data. Therefore, the results of previous studies were not able to confirm the relationship between circulating Fetuin-B and IR. In the current study, we conducted a cross-sectional study with relatively large sample size and multiple intervention studies. To avoid various interference factors, we optimized the grouping of experimental individuals. Young control and PCOS women were recruited for this study to compensate for the effects of age-related IR and gender. In addition, to prevent the impact of confounding factors such as the course of disease and drugs, only newly diagnosed PCOS women were included in this study.

The main findings of this study were as follows: (1) serum Fetuin-B was significantly increased in women with PCOS; (2) in obese women with PCOS, circulating Fetuin-B levels were higher than those in lean women with the disease; (3) serum Fetuin-B was associated with the indicators of glucose and lipid metabolism, IR, and inflammatory marker; (4) under physiological or IR conditions, glucose and insulin could exert different effects to increase production and/or secretion of Fetuin-B *in vivo*; (5) an acute increase of serum FFA levels reduced circulating Fetuin-B concentration; and (6) GLP-1RA treatment resulted in a significant reduction in serum Fetuin-B levels.

Here, we revealed serum Fetuin-B was markedly increased in females with PCOS compared to that in controls. In obese women with PCOS, circulating Fetuin-B levels were higher than those in lean women, suggesting an essential association of Fetuin-B with obesity. These results were similar to those noted in previous publications regarding other metabolic diseases, such as T2DM, NAFLD, and GDM [11, 12, 15, 21]. In addition, when our manuscript was being submitted, Adamska et al. published a pilot study that found that the serum Fetuin-B concentration in PCOS women was significantly higher than that in healthy controls, which was consistent with our results [22]. However, unlike the current study, the previous study was a small sample observational study. It did not investigate the direct effect of metabolic disorders and IR on circulating Fetuin-B, nor did it dynamically observe the changes of circulating Fetuin-B levels with the increase of insulin sensitivity. Therefore, the previous study was not adequately powered to demonstrate the association of Fetuin-B with IR in humans and did not use state-of-the-art methodology.

Furthermore, a study in streptozotocin-induced diabetic rats found that circulating levels of Fetuin-B was reduced relative to control rats [23]. We speculate that the difference may be due to the difference in species or experimental conditions and methods. Nevertheless, our data, as well as that of others, suggest a potential clue between Fetuin-B and the development of metabolic disorder and IR. The source of increased Fetuin-B in women with PCOS needs to be established in future experiments.

Like previously reported data regarding middle-aged and elderly adults and patients with NAFLD and/or T2DM [12, 13], our data for women with PCOS also showed that circulating Fetuin-B was associated with the indicators of glucose and lipid metabolism as well as IR, such as blood glucose, TG, and HOMA-IR. Interestingly, serum TNF*α* levels in PCOS women were significantly increased and positively correlated with serum Fetuin-B. These results imply that there is a chronic low-grade inflammatory state in women with PCOS, and changes in serum Fetuin-B may be related to inflammatory reactions. However, Meex et al. found that Fetuin-B upregulation in mice was due to an inflammatory status and not related to some biomarkers of obesity, IR, inflammation, and dyslipidemia in their study populations [10]. Thus, it is of great interest to further explore the relationship between circulating Fetuin-B and metabolic disorders and IR and to address the exact pathophysiological mechanisms by which Fetuin-B is increased in metabolic diseases.

Considering the relationship between circulating Fetuin-B and glucose and insulin, it may be modulated by glucose and/or insulin. To answer this question, we conducted an OGTT (both hyperglycemia and hyperinsulinemia) to study the impacts of nutritional status and hormone (insulin) on serum Fetuin-B *in vivo*. We provided important evidence for the dynamic alteration of Fetuin-B in response to a glucose challenge in healthy females and those with PCOS. Increased blood glucose and insulin levels led to a marked increase in serum Fetuin-B in control women rather than in PCOS women. The results from the OGTT suggest that in healthy women, hyperglycemia and/or hyperinsulinemia can stimulate the secretion and release of Fetuin-B *in vivo*. This scenario may also show that the body's response to hyperglycemia and hyperinsulinemia is different in both IR and non-IR states.

The EHC measured insulin's ability to stimulate glucose uptake in muscle and adipose tissue and inhibit lipolysis in adipose tissue [24]. With the EHC, individuals with PCOS have been reported to exhibit IR, as seen in obese women with hyperandrogenism, in whom plasma FFA levels are correlated with IR [25, 26]. During the EHC, blood glucose was controlled in the normal range, and exogenous insulin infusion led to an increase in insulin level. Therefore, a euglycemic-hyperinsulinemic state was established *in vivo*. This state allowed us to observe the regulation of Fetuin-B by hyperinsulinemia without being affected by blood glucose. Surprisingly, hyperinsulinemia led to a marked increase in circulating Fetuin-B levels in PCOS subjects, but not in control women. These results suggest that in healthy women, increased insulin levels did not stimulate the secretion and release of Fetuin-B *in vivo*. Therefore, the increased Fetuin-B level during the OGTT was mainly due to hyperglycemia stimulating its secretion. Therefore, blood glucose was the main factor affecting circulating Fetuin-B in healthy women, while in women with PCOS, hyperinsulinemia and IR were the main factors to determine circulating Fetuin-B levels. These results also suggest that under certain physiological or IR conditions, glucose and insulin can exert different effects on hepatocytes to increase production and/or secretion of Fetuin-B. However, in physiological or IR states, the mechanism underlying the response of circulating Fetuin-B to elevated blood glucose or insulin is unclear. A follow-up study is needed to illustrate this phenomenon.

It has been well known that elevated FFA leads to whole-body IR and adiposity [27]. Hyperlipidemia and hyperinsulinemia are two characteristic features of obesity and may contribute to the IR-associated elevation of circulating Fetuin-B in obese women and those with PCOS. Lipid infusion is known to increase serum FFA and TG and lead to acute IR *in vivo* [28]. To investigate the impact of an acute elevation of FFA and an acute IR induced by lipid infusion on circulating Fetuin-B, a 4 h lipid infusion and the EHC were simultaneously performed in healthy individuals. Interestingly, an acute increase of serum FFA levels and TG by lipid infusion reduced circulating Fetuin-B levels, suggesting that acute elevated FFA levels might be the main factor for affecting circulating Fetuin-B. In women with PCOS and individuals with lipid-induced IR, the reason for the bidirectional changes in circulating Fetuin-B levels was unclear. We speculate that in women with PCOS, long-term chronic IR may stimulate the increase of Fetuin-B synthesis and release *in vivo*. However, under an acute IR state induced by short-term serum FFA elevation, Fetuin-B release might be inhibited.

Antidiabetes drugs, such as metformin and thiazolidinediones (TZDs), have been widely used to treat women with PCOS [29–31]. In addition, GLP-1RA has been used to treat women with PCOS in a few small sample studies, demonstrating weight loss and improved IR, but none investigated the impact on IR-related cytokines [29, 30, 32]. In previous studies, GLP-1RAs have been reported to regulate some adipokine release, such as adiponectin and visfatin [33, 34]. In the current study, we found that in women with PCOS, six months of GLP-1RA treatment resulted in a significant reduction in serum Fetuin-B levels, which was accompanied by ameliorated glucose metabolism and insulin sensitivity as indicated by elevated *M* values. This observation further suggests that Fetuin-B is related to IR and demonstrates another beneficial role of GLP-1RA in regulating the secretion and release of cytokines *in vivo*. This raises the possibility that GLP-1RA might have an independent impact on regulating Fetuin-B release from the liver. However, the relationship between GLP-1RA and Fetuin-B is not clear. Our findings provide new clues for future research.

Nonetheless, these data highlight that GLP-1RA treatment of PCOS and other metabolic diseases can regulate circulating cytokines related to IR. These results should alert researchers who are working on Fetuin-B biology to notice this phenomenon. Furthermore, these results may be replicated with other anti-IR therapies.

Our data had some shortcomings: (1) Our research was made up of a Chinese population. Therefore, it was necessary to repeat the study with other races. (2) The nature of the cross-sectional study did not allow us to infer a causal relationship between elevated Fetuin-B concentration and the occurrence of PCOS. (3) In our and other studies, the concentration range of Fetuin-B was slightly different, which might be due to differences in the characteristics of the studied population. (4) Because of the design of the study, it was impossible to study circulating Fetuin-B levels longitudinally at different time points during the disease. In addition, all samples were measured in a single laboratory. Nonetheless, we believe that this study still provides sufficient evidence for the association of Fetuin-B with IR and PCOS and can attract the attention of other researchers.

## 5. Conclusions

Our results revealed that serum Fetuin-B levels were elevated in PCOS women compared with control women and were related to glucose metabolism and IR. Using multiple interventions, we showed that Fetuin-B levels in healthy women were affected by blood glucose levels, while in IR state, circulating Fetuin-B was affected by hyperinsulinemia. Importantly, we further found that GLP-1RA administration markedly decreased circulating Fetuin-B concentrations in women with PCOS, possibly through increased insulin sensitivity, or increased Fetuin-B synthesis and secretion. Therefore, the measurement of Fetuin-B concentration in clinical research can be used as a simple biomarker for screening metabolic disorders and IR in PCOS women. However, the physiological and pathological implications of these findings need to be further studied.

## Figures and Tables

**Table 1:** Main clinical features and serum Fetuin-B concentrations in PCOS and healthy women.

Variable	PCOS (*n* = 257)	Controls (*n* = 141)	*p* value
Age (years)	26.5 ± 3.8	26.3 ± 2.1	NS
BMI (kg/m^2^)	25.8 ± 4.6	20.2 ± 2.3	<0.01
FAT (%)	36.7 ± 7.6	26.5 ± 5.4	<0.01
WHR	0.87 ± 0.07	0.79 ± 0.06	<0.01
SBP (mmHg)	116.4 ± 12.3	108.1 ± 8.5	<0.01
DBP (mmHg)	74.6 ± 9.8	71.7 ± 9.0	<0.01
TC (mmol/L)	4.47 ± 1.08	3.88 ± 0.88	<0.01
TG (mmol/L)	1.81 ± 1.28	0.99 ± 0.66	<0.01
HDL-C (mmol/L)	1.26 ± 0.99	1.29 ± 0.38	NS
LDL-C (mmol/L)	2.66 ± 0.78	2.20 ± 0.76	<0.01
FFAs (*μ*mol/L)	0.55 ± 0.21	0.55 ± 0.27	NS
FBG (mmol/L)	5.39 ± 0.76	4.53 ± 0.46	<0.01
2 h-BG (mmol/L)	7.82 (6.33-9.25)	5.43 (4.71-6.32)	<0.01
FIns (mU/L)	18.42 (11.34-27.34)	7.12 (5.96-8.72)	<0.01
2 h-Ins (mU/L)	150.8 (99.1-249.6)	41.9 (24.3-68.7)	<0.01
*M* value (mg/kg/min)	4.77 ± 2.13	10.18 ± 2.55	<0.01
^§^ *M* value	5.21 ± 0.13	9.14 ± 0.21	<0.01
HOMA-_IR_	4.38 (2.64-6.95)	1.41 (1.15-1.81)	<0.01
^§^HOMA-_IR_	5.67 ± 0.60	2.90 ± 0.89	<0.05
AUCi (mU × h/L)	274.6 (194.6-397.5)	111.1 (73.0-146.6)	<0.01
AUCg (mmol × h/L)	17.12 (14.82-18.90)	11.98 (10.92-13.89)	<0.01
Fetuin-B (mg/L)	8.40 ± 5.02	6.22 ± 4.84	<0.01
^§^Fetuin-B	7.65 ± 0.32	6.72 ± 0.59	<0.05
DHEA-S (*μ*g/dL)	278.2 ± 134.5	228.5 ± 108.4	<0.01
LH (IU/L)	8.20 ± 5.00	4.59 ± 2.51	NS
FSH (IU/L)	6.36 ± 1.91	7.96 ± 1.93	<0.01
Estradiol (pg/mL)	44.2 (33.7-57.8)	44.4 (29.0-63.9)	NS
Prolactin (*μ*g/L)	14.46 ± 8.61	16.02 ± 8.08	NS
Prog (nmol/L)	1.67 (1.04-2.58)	2.18 (1.41-3.12)	NS
SHBG (nmol/L)	35.2 (24.9-49.5)	61.3 (77.6-45.3)	<0.01
TEST (nmol/L)	2.08 ± 0.82	1.67 ± 0.77	<0.01
FAI	6.99 ± 5.31	3.29 ± 2.77	<0.01

BMI: body mass index; FAT (%): the percentage of body fat; WHR: waist-to-hip ratio; SBP: systolic blood pressure; DBP: diastolic blood pressure; TC: total cholesterol; TG: triglyceride; HDL-C: high-density lipoprotein cholesterol; LDL-C: low-density lipoprotein cholesterol; FFAs: free fatty acids; FBG: fasting blood glucose; 2 h-BG: 2 h blood glucose after glucose overload; FIns: fasting insulin; 2 h-Ins: 2 h insulin after glucose overload; HOMA-_IR_: HOMA-insulin resistance index; AUCi: the area under the curve for insulin; AUCg: the area under the curve for glucose; free androgen index (FAI) = TEST (nmol/L)/SHBG (nmol/L) × 100. LH: luteinizing hormone; FSH: follicle-stimulating hormone; DHEA-S: dehydroepiandrosterone-sulfate; SHBG: sex hormone-binding globulin; TEST: testosterone; Prog: progesterone. Values were given as means ± SD or median (interquartile Range). Values were given as means ± SD or median (interquartile Range). ^§^Mean ± SE by a general linear model with adjustment of age and BMI.

**Table 2:** The results of linear regression analysis of variables associated with circulating Fetuin-B levels in the study population.

Variable	Fetuin-B		Age-and WHR-adjusted		Multivariate	
	*r*	*p*	*r*	*p*	*β*	*p*
Age (year)	0.03	NS				
BMI (kg/m^2^)	0.16	<0.01				
WHR	0.16	<0.01	0.06	NS	0.21	<0.05
FAT (%)	0.146	<01	0.07	NS		
SBP (mmHg)	0.14	<0.01	0.10	NS		
DBP (mmHg)	0.07	NS	0.03	NS		
TC (mmol/L)	0.07	NS	-0.03	NS		
TG (mmol/L)	0.17	<0.01	0.09	NS		
HDL-C (mmol/L)	-0.08	NS	-0.03	NS		
LDL-C (mmol/L)	0.12	<0.05	0.06	NS		
FFAs (*μ*mol/L)	0.08	NS	-0.09	NS		
FBG (mmol/L)	0.18	<0.01	0.20	<0.01		
2 h-BG (mmol/L)	0.16	<0.01	0.07	NS		
FIns (mU/L)	0.21	<0.01	0.22	<0.01		
2 h-Ins (mU/L)	0.16	<0.01	0.06	NS		
HOMA-_IR_	0.21	<0.01	0.25	<0.01		
AUCi (mU × h/L)	0.17	<0.01	0.05	NS		
AUCg (mmol × h/L)	0.20	<0.01	0.26	<0.01		
*M* value (mg/kg/min)	-0.24	<0.01	-0.18	<0.01	-0.400	<0.01
DHEA-S (*μ*g/dL)	-0.03	NS	-0.10	NS		
LH (IU/L)	0.09	NS	0.04	NS		
FSH (IU/L)	-0.16	<0.01	-0.11	<0.05		
Estradiol (pg/mL)	-0.04	NS	-0.03	NS		
Prolactin (*μ*g/L)	-0.107	<0.05	-0.05	NS		
Prog (nmol/L)	-0.068	NS	0.05	NS		
SHBG (nmol/L)	-0.057	NS	0.10	NS		
TEST (nmol/L)	-0.003	NS	-0.08	NS		
FAI	0.026	NS	-0.03	NS		
TNF*α* (ng/L)	0.212	<0.01	0.04	NS		

In multiple linear stepwise regression analysis, values included for analysis were age, BMI, WHR, SBP, DBP, HOMA-IR, FFA, TG, TC, HDL-C, LDL-C, FAT%, DHEA-S, LH, FSH, Prog, and FAI.

## Data Availability

The data used to support the findings of this study are available from the corresponding author upon request.

## Supplementary Materials

Supplementary materials We have prepared supplementary material and have submitted it along with our manuscript, including material and methods, Table S1-S3, and Figure S1-S3. Table S1: association of circulating Fetuin-B with PCOS in fully adjusted models. Table S2: row mean scores and Cochran-Armitage trend test of the impact of circulating Fetuin-B levels on PCOS and IR. Table S3: metabolic features and hormone parameters pre- and posttreatment with GLP-1RA in PCOS women. Figure S1: study flow chart. Figure S2: schematic diagram for GLP-1RA study. Arrows indicate blood sample collection for the measurements of Fetuin-B, biochemical, and hormonal parameters. After admission, the liraglutide dose was increased weekly by 0.6 mg/d until participants were using 1.8 mg once daily. 75 g OGTT and EHC are performed at 8:00 AM on the indicated days. Figure S3: ROC curve analyses for the prediction of (a) PCOS and (b) IR, according to circulating Fetuin-B levels.
