# Supplementary material for: Elevated Circulating Fetuin-B Levels Are Associated with Insulin Resistance and Reduced by GLP-1RA in Newly Diagnosed PCOS Women
Source: Mediators Inflamm. 2020 Sep 30;2020:2483435. doi: 10.1155/2020/2483435 (PMC7545451; doi:10.1155/2020/2483435)
Supplement: Supplementary materials — We have prepared supplementary material and have submitted it along with our manuscript, including material and methods, Table S1-S3, and Figure S1-S3. Table S1: association of circulating Fetuin-B with PCOS in fully adjusted models. Table S2: row mean scores and Cochran-Armitage trend test of the impact of circulating Fetuin-B levels on PCOS and IR. Table S3: metabolic features and hormone parameters pre- and posttreatment with GLP-1RA in PCOS women. Figure S1: study flow chart. Figure S2: schematic diagram for GLP-1RA study. Arrows indicate blood sample collection for the measurements of Fetuin-B, biochemical, and hormonal parameters. After admission, the liraglutide dose was increased weekly by 0.6 mg/d until participants were using 1.8 mg once daily. 75 g OGTT and EHC are performed at 8:00 AM on the indicated days. Figure S3: ROC curve analyses for the prediction of (a) PCOS and (b) IR, according to circulating Fetuin-B levels. [file 2483435.f1.docx]

**Supplementary material**

**Material and Methods**

***Cross-sectional study***

PCOS diagnosis needs to meet at least two of the following three criteria: 1) oligo- amenorrhea or chronic anovulation; 2) clinical and/or biochemical hyperandrogenism; 3) ultrasound appearance of polycystic ovaries, after exclusion of other known causes of hyperandrogenemia and ovulatory dysfunction, including 21-hydroxylase deficiency, congenital adrenal hyperplasia, Cushing’s syndrome, thyroid disease, and hyperprolactinemia. Women with PCOS were newly diagnosed and had not been treated with any agents. In the study populations, those with age > 35 years old, thyroid disease, tumor, diabetes, hypertension and other organic diseases are excluded. All women in the study were in the follicular phase. In the past three months, none of the participants took hormones or drugs that affected insulin sensitivity. Written informed consent was given to all participants. This study was conducted by the Declaration of Helsinki and supported by the ethical committee of Chongqing Medical University.

***Biochemical parameters and hormone measurements***

Blood glucose was determined by the glucose oxidase method. Insulin levels were measured by ELISA using a commercial kit. FFAs were examined with a commercial kit (Randox Laboratories Ltd., Antrim, UK, FA115). Total cholesterol (TC), low-density lipoprotein cholesterol (LDL-C), high-density lipoprotein cholesterol (HDL-C), and triglyceride (TG) were measured enzymatically using an automatic biochemical instrument. Serum luteinizing hormone (LH), follicle-stimulating hormone (FSH), prolactin (PRL), estradiol, and progesterone (Prog) were measured by electrochemiluminescence. Serum testosterone (TEST) was examined by RIA. An automated analyzer was used to measure serum dehydroepiandrostenedione sulfate (DHEA-S) and sex hormone-binding globulin (SHBG) [19].

***Anthropometric examination***

Fourteen hours after fasting, all women took part in a series of anthropometry and body composition examinations, including waist circumference (WC), body weight, and height. BMI was calculated as weight divided by height squared. The percentage of body fat (FAT %) was measured by bioelectrical impedance (BIA-101; RJL Systems). The ratio of waist-to-hip (WHR) was calculated by the same observer, as previously reported [19]. After a rest period of 10 min, blood pressure (BP) was examined in the sitting position. The average of 3 examinations was calculated.

***Oral glucose tolerance test (OGTT)***

At 8 a.m., after 8-10 hours of fasting, the OGTT test was performed in all study populations. These participants ingested 75 g glucose, and blood was drawn at indicated time points (0, 30, 60, and 120 min) for the measurement of glucose, insulin, and Fetuin-B.

**Table S1** Association of circulating Fetuin-B with PCOS in fully adjusted models

| Model adjustment | PCOS | | |
| --- | --- | --- | --- |
|  | OR | 95% CI | *P* |
| Age  Age, BMI  Age, BMI, WHR | 1.106 | 1.053-1.162 | < 0.001 |
|  | 1.080 | 1.021-1.114 | < 0.01 |
|  | 1.078 | 1.017-1.143 | < 0.05 |
| Age, BMI, WHR, FAT% | 1.075 | 1.013-1.141 | < 0.05 |
| Age, BMI, WHR, FAT%, BP | 1.072 | 1.009-1.138 | < 0.05 |
| Age, BMI, WHR, FAT%, BP, lipid profile | 1.059 | 0.985-1.138 | NS |

Results of multivariate binary logistic regression analysis are presented as the odds ratio (OR) for having an increased PCOS status in circulating fetuin-B. Lipid profile includes TC, TG, LDL-C, HDL-C, and FFAs.

**Table S2** Row mean scores and Cochran–Armitage trend test of the impact of circulating Fetuin-B levels on PCOS and IR.

|  | PCOS | | IR | |
| --- | --- | --- | --- | --- |
|  |  | *P*-value | χ² | *p*-value |
| ROW Mean Scores Test | 71.323 | < 0.001 | 81.731 | < 0.001 |
| Cochran-Armitage Test | 4.492 | < 0.001 | 3.923 | < 0.001 |

**Table S3** Metabolic features and hormone parameters pre- and post-treatment with GLP-1RA in PCOS women

| Variable | Baseline | Post-treatment  3 months | Post-treatment  6 months |
| --- | --- | --- | --- |
| BMI (kg/m^2^) | 28.9 ± 3.0 | 26.8 ± 2.9^**^ | 26.2 ± 3.3^**^ |
| FAT (%) | 42.1 ± 7.3 | 37.1 ± 4.2^**^ | 36.6 ± 5.1^**^ |
| WHR | 0.89 ± 0.06 | 0.88 ± 0.06 | 0.88 ± 0.06 |
| SBP (mmHg) | 116.2 ± 10.9 | 112.7 ± 9.4 | 114.6 ± 12.4 |
| DBP (mmHg) | 75.0 ± 9.3 | 76.0 ± 9.9 | 74.7 ± 9.6 |
| TC (mmol/L) | 4.79 ± 0.84 | 4.14 ± 0.83^**^ | 4.24 ± 0.81^**^ |
| TG (mmol/L) | 2.02 ± 0.82 | 1.54 ± 0.67^**^ | 1.36 ± 0.67^**^ |
| HDL-C (mmol/L) | 1.17 ± 0.26 | 1.16 ± 0.19 | 1.21 ± 0.59 |
| LDL-C (mmol/L) | 2.98 ± 0.75 | 2.52 ± 0.75 | 2.51 ± 0.75 |
| FFAs (µmol/L) | 0.53 ± 0.18 | 0.47 ± 0.15 | 0.49 ± 0.41 |
| FBG (mmol/L) | 5.51 ± 0.76 | 5.23 ± 0.43 | 5.18 ± 0.39^*^ |
| 2h-BG (mmol/L) | 9.70 ± 3.88 | 7.85 ± 2.37^*^ | 7.32 ± 2.02^**^ |
| FIns (mU/L) | 28.4 ± 14.1 | 18.9 ± 8.8^**^ | 17.4 ± 9.7^**^ |
| 2h-Ins (mU/L) | 258.2 ± 195.2 | 211.6 ± 144.1 | 173.9 ± 149.0^*^ |
| M-value | 3.46 ± 0.84 | 4.78 ± 1.64^**^ | 4.97 ± 1.76^**^ |
| HOMA-_IR_ | 7.11 ± 4.00 | 4.45 ± 2.20^**^ | 4.06 ± 2.35^**^ |
| AUCi (mU×h/L) | 410.6 ± 260.1 | 343.3 ± 185.8 | 312.9 ± 170.5^*^ |
| AUCg (mmol×h/L) | 19.0 ± 4.3 | 16.6 ± 3.6^**^ | 16.1 ± 2.8^**^ |
| DHEA-S (µg/dL) | 274.8 ± 156.2 | 244.5 ± 139.9 | 276.5 ± 132.7 |
| LH (IU/L) | 6.10 ± 3.62 | 6.19 ± 3.27 | 6.65 ± 3.97 |
| FSH (IU/L) | 6.09 ± 1.52 | 5.74 ± 2.12 | 5.88 ± 1.83 |
| LH/FSH ratio | 1.03 ± 0.63 | 1.25 ± 0.82 | 1.21 ± 0.83 |
| Estradiol (pg/ml) | 41.5 (30.8-54.6) | 45.00 (33.0-61.1) | 43.35 (33.9-61.6) |
| Prolactin (µg/L) | 14.4 ± 7.5 | 18.8 ± 11.4^*^ | 19.2 ± 7.8^**^ |
| Prog (nmol/L) | 1.46 (0.95-2.07) | 1.16 (0.77-2.42) | 1.21 (0.86-2.55) |
| SHBG (nmol/L) | 31.1 (21.3-45.8) | 34.3 (26.3-42.7) | 37.7 (26.4-53.2) |
| TEST (nmol/L) | 2.01 ± 0.75 | 1.47 ± 0.49^**^ | 1.48± 0.47^**^ |
| FAI | 5.48 (4.06-9.74) | 4.08 (2.93-6.13)^*^ | 3.82 (2.64-5.45)^*^ |
| Fetuin-B (mg/L) | 9.22 ± 4.15 | 8.23 ± 2.87 | 7.27 ± 2.50^*^ |

Data are means ± standard deviation or median (interquartile range). * *p* < 0.05, ** *p* < 0.01 *vs.* Baseline.


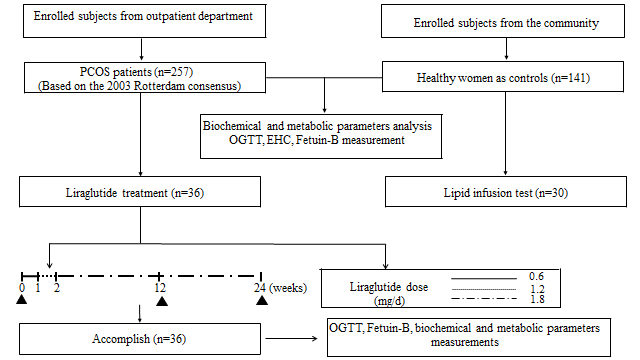


**Figure S1** Study flow chart.


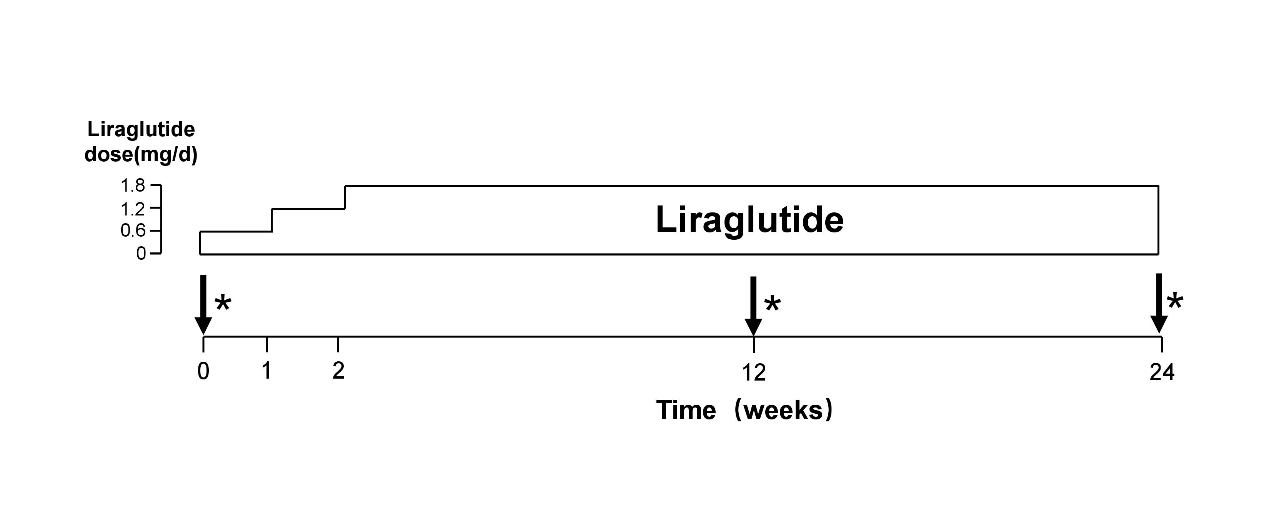


**Figure S2** Schematic diagram for GLP-1RA study. Arrows indicate blood samples collection for the measurements of Fetuin-B, biochemical and hormonal parameters. After admission, the liraglutide dose was increased weekly by 0.6 mg/d until participants were using 1.8 mg once daily. 75g OGTT and EHC are performed at 8:00 AM on the indicated days.


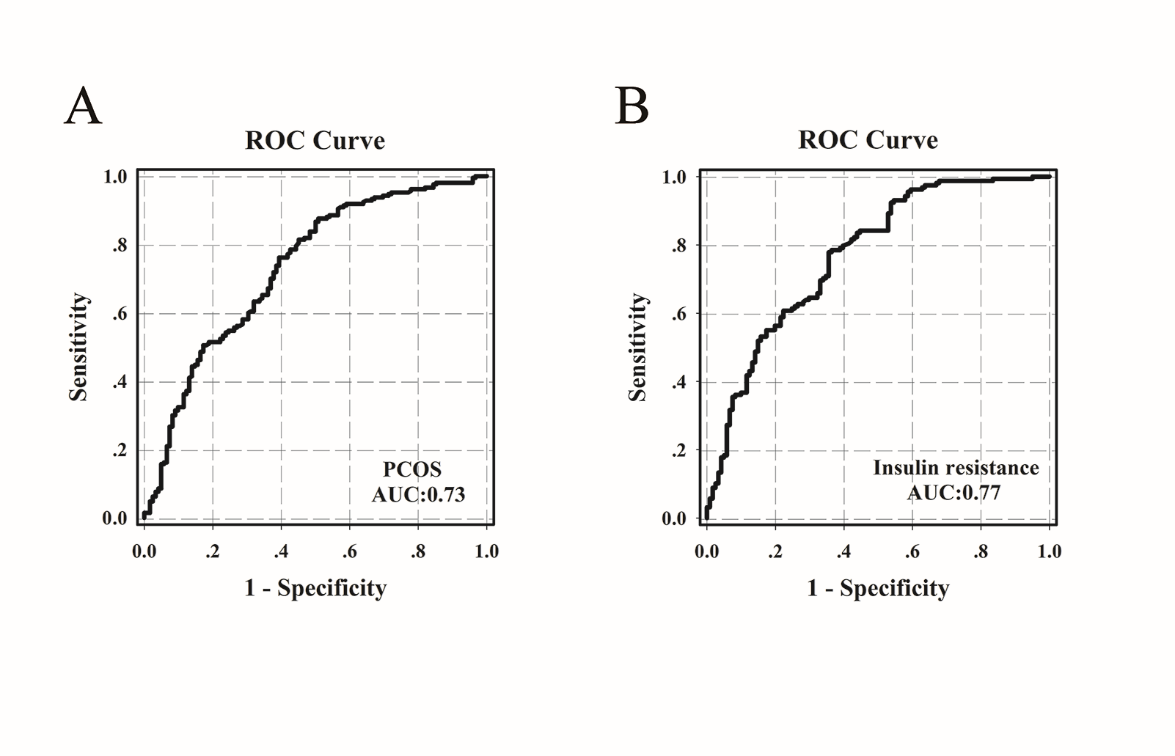


**Figure S3** ROC curve analyses for the prediction of **(A)** PCOS and **(B)** IR, according to circulating Fetuin-B levels.
